# Dual-Modality Imaging of the Human Finger Joint Systems by Using Combined Multispectral Photoacoustic Computed Tomography and Ultrasound Computed Tomography

**DOI:** 10.1155/2016/1453272

**Published:** 2016-09-27

**Authors:** Yubin Liu, Yating Wang, Zhen Yuan

**Affiliations:** Bioimaging Core, Faculty of Health Sciences, University of Macau, Macau

## Abstract

We developed a homemade dual-modality imaging system that combines multispectral photoacoustic computed tomography and ultrasound computed tomography for reconstructing the structural and functional information of human finger joint systems. The fused multispectral photoacoustic-ultrasound computed tomography (MPAUCT) system was examined by the phantom and* in vivo* experimental tests. The imaging results indicate that the hard tissues such as the bones and the soft tissues including the blood vessels, the tendon, the skins, and the subcutaneous tissues in the finger joints systems can be effectively recovered by using our multimodality MPAUCT system. The developed MPAUCT system is able to provide us with more comprehensive information of the human finger joints, which shows its potential for characterization and diagnosis of bone or joint diseases.

## 1. Introduction

Photoacoustic computed tomography (PACT), also called optoacoustic computed tomography, is able to visualize the structural and functional information about biological tissues with excellent acoustic resolution and high optical contrast [1–3]. PACT is concerned with an inverse problem, in which the spatial distribution of the optical absorption can be reconstructed based on the measured acoustic pressures along the tissue boundary. To date, PACT has been extensively explored for the detection of breast cancer, for probing brain functioning in small animals, and for assessing vascular and skin diseases [4–6]. In addition, PACT is able to recover the tissue physiological properties by using spectrally resolved photoacoustic (PA) measurements. The multispectral PACT (MPACT) can reveal spatially resolved physiological and molecular information by using the known spectral characteristics of specific chromophores [7–10]. In addition, ultrasound computed tomography (USCT) also has the capability to recover the structure information of biological tissues, which can be complementary to the MPACT that has demonstrated its advantages for functional imaging. The final aim of the present study is to develop and validate a dual-modality imaging technique, namely, MPAUCT that combines MPACT and USCT for* in vivo* imaging the progression of finger joint osteoarthritis and psoriatic arthritis.

Osteoarthritis (OA) is the most common arthritic condition worldwide and is estimated to affect nearly 60 million Americans. The prime features of OA include the progressive degeneration of articular cartilage, subchondral bone remodeling, osteophyte formation, and a variable degree of synovitis [11, 12]. In addition, psoriasis is a common hyperkeratotic skin disease that affects 7.5 million Americans [13, 14]. About 10 percent to 30 percent of people with psoriasis also develop psoriatic arthritis (PA). PA is an inflammatory arthritis associated with psoriasis that affects peripheral synovial joints and entheses and the axial skeleton [13, 14]. Interestingly, recent phantom and clinical studies show that PACT with/without USCT has been performed as a potential and effective tool for imaging human finger joints [15–19]. For instance, Wang et al. developed a dual-modality PACT-US system that could identify the bones and delineate tendons from other soft tissues, though they could not recover most of the blood vessels in the finger joint [15]. In addition, Xi and Jiang demonstrated a three-dimensional PACT system that utilized cylindrical scanning in data collection and virtual-detector concept in image reconstruction to generate a high-resolution image of the human finger joint [16]. Likewise, this imaging technique could not identify most of the soft tissues and blood vessels. Furthermore, a group in University of Twente conducted a PACT investigation on a healthy human finger joint in order to image the blood vessels with a focus on vascularity across the interphalangeal joints [17, 18]. In this study, we present a novel homemade MPAUCT system for dual-modality imaging of human finer joints systems. The multispectral and multimodality imaging methods are able to recover the different structural and functional information within the finger joints systems including the bones, the skins and the subcutaneous tissues, the tendons, and the majority of the blood vessels. More importantly, the mutual complementation of MPACT and USCT will provide us with more comprehensive information in studying human finger joint systems, which shows the potential for the characterization and diagnosis of finger joint diseases such as osteoarthritis and psoriatic arthritis.

## 2. Materials and Methods

### 2.1. The Developed MPAUCT Imager for Finger Joint Imaging

**Figure 1:**
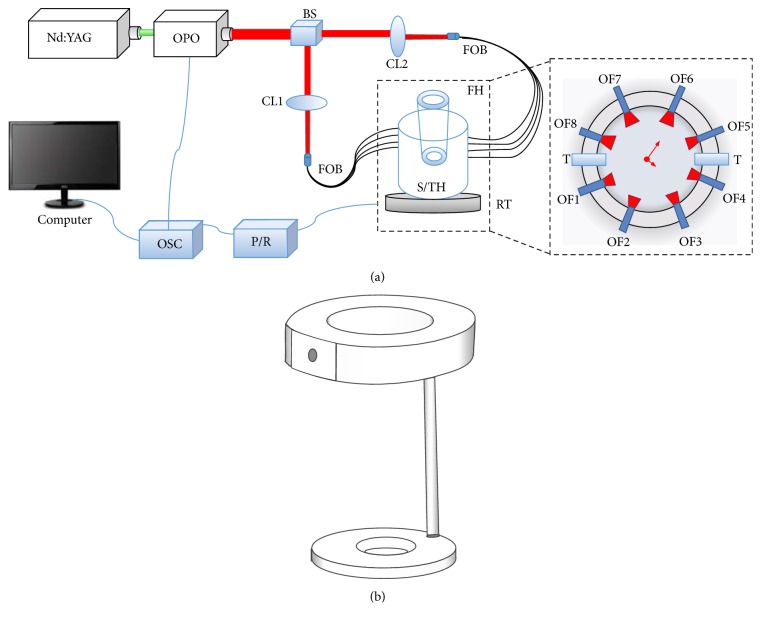
(a) Experimental configuration of the MPAUCT dual-modality imaging system. OPO: optical parametric oscillator, OSC: oscilloscope, P/R: pulser/receiver, BS: beam splitter, CL: convex lens, FOB: fiber optics bundle, FH: finger holder, S/TH: sink/transducer holder, RT: rotary table, T: transducer, and OF: optical fiber. (b) Schematic of the finger holder.

The schematic of the MPAUCT imaging system is shown in Figure 1(a). A pulsed light from OPO is pumped by the Nd:YAG laser (Surelite I-10, Continuum) with the wavelengths of 680~1064 nm, pulse durations of 5~10 ns, and frequency rate of 20 Hz. The laser from Nd:YAG has the wavelengths of 532 nm and 1064 nm, in which the laser at the wavelength of 532 nm is used for pumping the OPO crystal, and the OPO crystal can generate the wavelengths range within 680~1064 nm at different crystal angle.

The laser beam from OPO is separated into two parts that transport in different paths; then the two-light beams pass through convex lens 1 and convex lens 2 and finally are focused into two fiber optics bundles. The two paths delivered by two fiber optics bundles are further divided into 8 light beams (each beam has the energy density of 8 mJ/cm^2^) to illuminate the cross sections of the fingers/finger joints. The optical fiber bundles were custom-made silica fibers with one input fiber and four output fibers. In addition, the core diameter of each fiber is 600 um, the NA is 0.23, the transmission wavelengths range within 580~1200 nm, and the transmittance can research over 65%. The finger holder is fabricated by the aluminum alloy (Figure 1(b), which consists of three parts: two annuli with different sizes and one joint lever that connect the two annuli).

The 8 optical fibers and two unfocused ultrasound transducers (3.5 MHz central frequency; bandwidth range within 3.44~6.60 MHz; V309, Olympus NDT) are fastened on the holder. The holder and the sink are placed on the rotator. The pulser/receiver (5073PR, OLYMPUS) has the dual-mode with the functions of ultrasonic emission and reception, so the PA and US signals are finally received and amplified by the pulser/receiver apparatus. The output signals are shown on the oscilloscope with the average of 16 times and then stored on the computer for further data processing.

### 2.2. Delay-and-Sum Beam Forming Algorithm

To form the PA and US images of an object, an image reconstruction algorithm is required. A simple, yet very effective method is the delay-and-sum beam forming algorithm that is commonly used in radar signal processing. The image expression in the case of near-field for PA and US imaging can be stated as

(1) $$\begin{array}{c} S^{f}\left(\;t\;\right)=\frac{\sum_{i}w_{i}^{f}S_{i}\left(t+\delta_{i}^{f}\right)}{\sum_{i}w_{i}^{f}}, \end{array}$$

where *S* ^*f*^(*t*) is the image output at a particular focus point *f*, *S*_*i*_(*t*) is the time signal from *i*th receiver, *δ*_*i*_^*f*^ is the delay applied to this signal, and *w*_*i*_^*f*^ is an amplitude weighting factor, which is used to enhance the beam shape, to reduce side lobe effects, or to minimize the noise level. The summed signal is typically normalized to make the output independent of the actual set of transducers. The different is PA signals processing without any deconvolution and filtering, whereas the ultrasound images reconstruction used is with the filtering and deconvolution.

### 2.3. Phantom and* In Vivo* Experimental Tests

**Figure 2:**
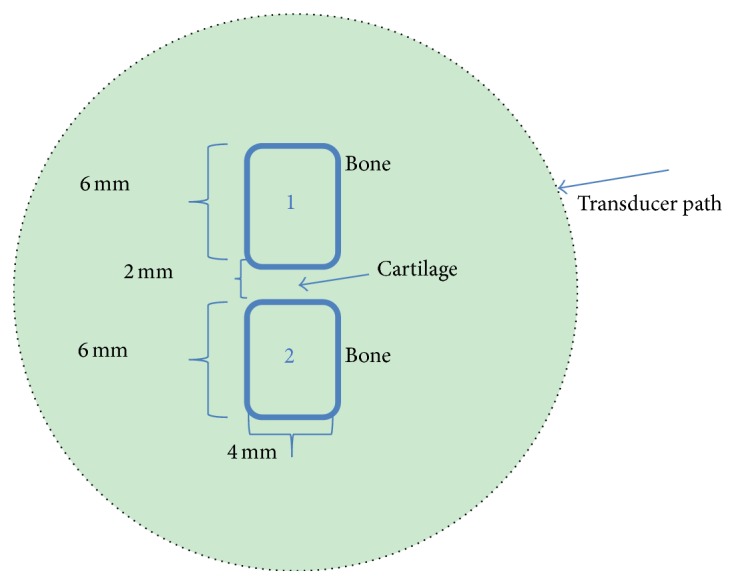
Schematic of the test geometry for the finger joint phantom.

In this section, we conducted phantom and* in vivo* tests using our homemade MPAUCT system. For the phantom test as shown in Figure 2, a cylindrical solid phantom was used as the background medium. And two cylindrical “bones” (the diameter: 4 mm; the height: 6 mm) were immersed into the phantom. The “cartilage” located between the two “bones” had the height size of 2 mm. The optical properties of the phantom and the “bones” were provided in Table 1. The “cartilage” was assumed to have the same optical properties with the background phantom. The background phantom materials utilized were composed of Intralipid as scatterer and India ink as absorber with agar powder (1-2%) for solidifying, whereas the two cylindrical bone targets also consisted of the same materials but had higher absorption and scattering contrasts compared to the background phantom [20]. In addition, the actual acoustic contrast can easily be accessed by using the following longitudinal velocities in Table 2 for the different tissues including the agar phantom we used.

For the* in vivo* experiment test, we reconstructed the proximal interphalangeal (PIP) finger joint of a healthy volunteer by using our MPAUCT imaging system. PA data along each cross-sectional plane (slice) of the PIP finger joints systems were acquired for the subject at the wavelength of 680 nm, 720 nm, 760 nm, and 800 nm. One set of PA data for each wavelength was taken at 240 positions with the angular step size of 1.5 degrees using two transducers with the central frequency of 3.5 MHz. The data acquisition and image reconstruction for each slice took about 2~3 minutes. The PA data and US data were acquired along the same slice so that we could easily compare the reconstruction information from MPACT images and US images and then generate the merged MPAUCT images.

## 3. Results and Discussion

**Figure 3:**
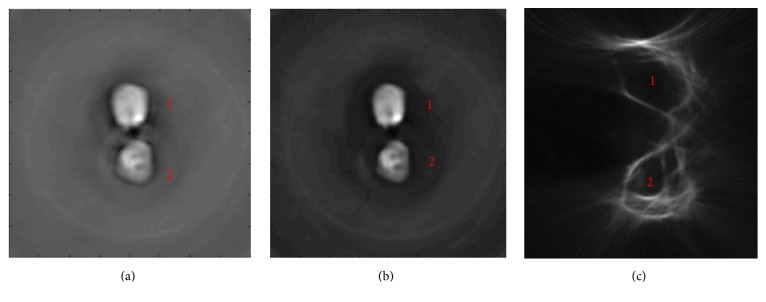
Reconstructed MPACT images of the finger joint phantom at the wavelength of 700 nm (a) and 760 nm (b). (c) Reconstructed USCT image of the finger joint phantom. The top is bone 1; the bottom is bone 2.

From the reconstruction results in Figures 3(a) and 3(b), we discovered that the “bones” were clearly identified for the finger joint phantom at the wavelengths of 700 nm and 760 nm by using the MPAUCT imaging system. Specifically, compared with the PACT images, we observed from the USCT findings in Figure 3(c) that there was a significant drop in the imaging contrast between the “bones” and the phantom. It is due to the fact that the acoustic contrast between them was much lower compared to the optical contrast. Interestingly, the USCT images also showed strong boundary effects compared to the MPACT images. In addition, it should be pointed out that the acoustic contrast is due to the difference of the mechanical properties including acoustic velocity or elastic modulus between the different media. In this study, the ultrasound image is poorly reconstructed for the phantom test because the acoustic contrast between the target (bones) and the background media is nearly equivalent to one.

**Figure 4:**
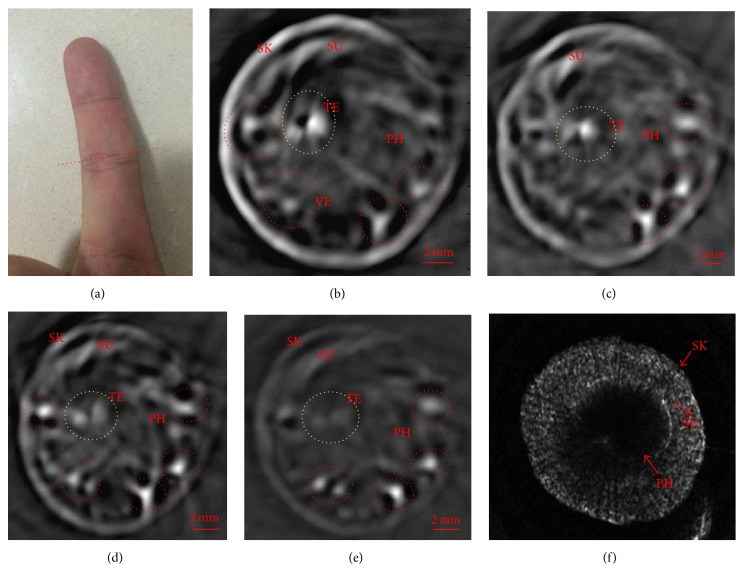
(a) The photography of the finger joint measurement configuration. (b)–(e) Reconstructed cross-sectional MPACT images of a human subject finger at the wavelengths of 680 nm, 720 nm, 760 nm, and 800 nm, respectively. (f) Reconstructed cross-sectional USCT image along the same plane of the same finger joint systems. SK: skin, SU: subcutaneous tissue, VE: blood vessels, TE: tendon, and PH: phalanx.

Figure 4 shows the MPACT images and USCT images of the human PIP finger joint systems recovered by using our MPAUCT imaging system. We discovered that the MPACT images along a cross-sectional slice of the PIP finger joint systems (marked with dotted red curve) were successfully recovered and plotted in Figures 4(b)–4(e) for the wavelength of 680 nm, 720 nm, 760 nm, and 800 nm, respectively. It was observed from Figure 4 that the soft tissues including skin, subcutaneous tissue, and the tendon were clearly identified for all the wavelengths, as shown in Figures 4(b)–4(e). More importantly, we found from Figures 4(b)–4(e) that the majority of the blood vessels were also recovered (marked by red dotted circles) for all the four wavelengths by using our dual-modal imaging system. By contrast, the hard tissue such as the phalanx was only photoacoustically recovered at the wavelength of 680 nm, whereas the phalanx images became blurred for the other three wavelengths due to the change of optical absorption of bone tissues at different wavelengths. For instance, the chromophores of the bones at different wavelengths show different optical absorption contrasts according to the optical absorption spectra [11]. In particular, our findings suggested that the identified structural information including the tendon showed good agreement with the previous results as well as the anatomy of finger joint system [11, 15–19].

Finally, Figure 4(f) displayed the reconstructed USCT image along the same slice of the MPACT images, where we discovered that the soft tissues including the skins and subcutaneous tissues were clearly recovered. What is more, the phalanx could be reconstructed with the higher contrast and higher resolution by USCT compared to that from the MPACT.

**Figure 5:**
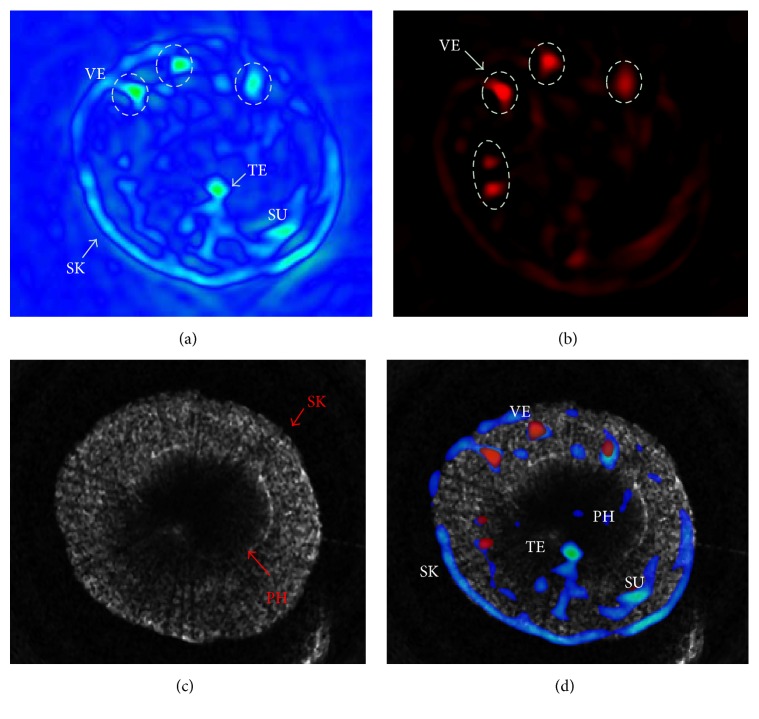
Reconstructed cross-sectional MPACT images of a human subject finger at the wavelengths of 720 nm (a) and 800 nm (b). (c) Reconstructed cross-sectional USCT image along the same plane of the same finger joint systems. (d) Coregistered MPAUCT images of the human finger joint systems generated by MPACT/USCT merging. SK: skin, SU: subcutaneous tissue, VE: blood vessels, TE: tendon, and PH: phalanx.

In summary, we have developed a homemade MPAUCT dual-modality imaging system that can effectively reconstruct the human finger joint systems including the skins, the blood vessels, the tendon, and the bone simultaneously. The preliminary results in Figure 5 generated from the* in vivo* test indicated that the MPAUCT system can be implemented with the same hardware and can exhibit both the optical and ultrasound contrast mechanisms. More importantly, the developed MPAUCT system is able to provide us with more comprehensive information of the human finger joints, which shows its potential for characterization and diagnosis of bone or joint diseases. Further investigations are warranted to use of high central frequency and focused transducers to improve the imaging resolution.

## Figures and Tables

**Table 1:** Optical properties used for the finger joint phantom test.

Wavelength (nm)	Optical properties	Background	Target 1	Target 2
700	Absorption coefficient (mm^−1^)	0.01	0.14	0.12
	Scattering coefficient (mm^−1^)	1	2	2

760	Absorption coefficient (mm^−1^)	0.01	0.143	0.122
	Scattering coefficient (mm^−1^)	1	2	2

**Table 2:** Average longitudinal velocities for the different phantoms [21].

Velocity	Agar	Porcine	Fat turkey breast	Bovine liver
vs (m/s)	1489.3	1501.6	1576.3	1590.0
